# Subliminal attention bias modification training in socially anxious individuals

**DOI:** 10.3389/fnhum.2013.00389

**Published:** 2013-07-23

**Authors:** Keren Maoz, Rany Abend, Nathan A. Fox, Daniel S. Pine, Yair Bar-Haim

**Affiliations:** ^1^School of Psychological Sciences, Tel Aviv UniversityTel-Aviv, Israel; ^2^Department of Human Development, University of MarylandCollege Park MD, USA; ^3^National Institute of Mental HealthBethesda, MD, USA; ^4^Sagol School of Neuroscience, Tel Aviv UniversityTel-Aviv, Israel

**Keywords:** social anxiety, stress vulnerability, attention bias modification, masking, subliminal

## Abstract

Anxious individuals demonstrate threat-related attention biases both when threat stimuli are presented within conscious awareness and when presented below awareness threshold. Nevertheless, attention bias modification (ABM) research has rarely utilized sub-awareness protocols in an attempt to modify attention patterns and reduce anxiety. Exploring the potential of subliminal ABM is of interest, as it may target attention processes related to anxiety that are distinct from those engaged by supraliminal ABM. Here we examined the effect of a subliminal ABM training protocol on levels of social anxiety and stress vulnerability. Fifty-one socially anxious students were randomly assigned to either ABM or placebo condition, and completed a pre-training assessment, four training sessions, a social stressor task, and a post-training assessment. Results indicate that the subliminal ABM used here did not induce detectable changes in threat-related attention from pre- to post-training as measured by two independent attention tasks. Furthermore, the ABM and placebo groups did not differ on either self-reported social anxiety post-training or state anxiety following stress induction. *Post-hoc* auxiliary analyses suggest that ABM may be associated with smaller elevations in state anxiety during the stressor task only for participants who demonstrate attention bias toward threat at baseline. Implications and future research directions are discussed.

## Introduction

Numerous studies across clinical and sub-clinical populations have found that anxious individuals demonstrate an attentional bias toward threat-related stimuli (Bar-Haim et al., 2007). This bias manifests even when threat stimuli are presented below awareness thresholds (Mathews and MacLeod, 1986; Mogg et al., 1993; van den Hout et al., 1995; Fox, 2002; Mogg and Bradley, 2002). Additional findings further suggest that threat biases causally affect stress vulnerability (MacLeod et al., 2002; Mathews and MacLeod, 2002; Eldar et al., 2008). Based on such observations, attention bias modification (ABM) treatments have started to emerge, exploring the potential of computerized tools to modify attention patterns and consequently reduce stress-vulnerability and anxiety (Koster et al., 2009; Bar-Haim, 2010; Hakamata et al., 2010; Beard, 2011; Hallion and Ruscio, 2011).

Clinical ABM trials indicate that training patients to attend away from threat reduces self-reported as well as clinically evaluated anxiety levels (Amir et al., 2009, 2011; Schmidt et al., 2009; Eldar et al., 2012). In addition, studies with non-clinical high-anxious participants show that ABM training typically reduces stress vulnerability in the face of lab-induced (Amir et al., 2008; Bar-Haim et al., 2011) or real-life (See et al., 2009) stressors. However, although many studies have utilized subliminal stimuli to measure preconscious threat-related attention biases and their associations to anxiety and stress vulnerability (MacLeod and Hagan, 1992; van den Hout et al., 1995; Fox et al., 2010), the vast majority of ABM studies to date have used supraliminal (i.e., consciously perceived) stimuli presentations to modify these biases.

Exploring the potential of subliminal ABM is of interest, as it may target a different layer of attention processes related to anxiety. In line with this idea, brain imaging and psychophysiology studies found distinct responses to readily-identifiable, as opposed to masked, subliminal threat-related stimuli in anxious relative to non-anxious individuals (Ohman and Soares, 1994; Etkin et al., 2004; Li et al., 2007; Tsunoda et al., 2008). These findings suggest that hypersensitivity to threat in anxious individuals may occur prior to conscious awareness. For example, Etkin et al. (2004) demonstrated that supraliminal and subliminal presentations of threat faces modulated neural activation in distinct regions of the amygdala. Specifically, subliminal presentations modulated activity in the basolateral region of the amygdala, and this activation was positively correlated with trait anxiety. Thus, subliminal ABM may provide an opportunity to intervene with anxiety-maintaining mechanisms that act very early in the processing stream.

To our knowledge only one study used subliminal presentations in the context of ABM. MacLeod et al. (2002; study 1) used a dot-probe task to train non-anxious students to attend either to threat or neutral words. A single-session protocol with 576 active training trials was used, in which half of the trials were presented subliminally and half were presented well within conscious awareness. In addition, 96 attention bias measurement trials were intermixed throughout the active training trials. Half of these measurement trials were subliminally presented and the other half were presented within conscious awareness. Following training, lower stress vulnerability was found in the group trained to attend away from threat relative to the group trained to attend toward threat. The results also indicated that attentional changes following training emerged only for consciously presented measurement trials and not for subliminal measurement trials. However, because all trial types (subliminal, supraliminal; training, measurement) were presented in a mixed fashion, conclusive inference on the specific effect of subliminal training was complicated. Moreover, as mentioned, this study included only non-anxious participants. It has been previously demonstrated that non-anxious individuals' attention is less reactive to subliminal threatening stimuli as compared to anxious individuals (Mathews and MacLeod, 1986; Mogg et al., 1993). This could suggest a possible explanation as to why no change in preconscious attention processes was demonstrated following training among these non-anxious subjects. Finally, the study by MacLeod et al. (2002) used word stimuli that might be less optimal than evolutionary-relevant threat, such as faces (Ohman and Mineka, 2001), for early threat-attention modification processes. Thus, it appears that more research is needed to explore the effects of subliminal ABM on anxiety and stress vulnerability among anxious individuals.

The aim of the current study was to examine the efficacy of a subliminal dot-probe ABM protocol on attention bias, anxiety levels, and stress vulnerability in a group of undergraduate students with high levels of self-reported social anxiety. We decided to focus on socially anxious individuals for several reasons: first, for methodological reasons, we wanted to keep our sample as homogenous as possible with respect to the nature of their anxiety. This enabled us to specify the stimuli and the stressful manipulation to the characteristics of this particular anxiety. Second, we selected this specific population because previous findings indicate ABM efficacy with supraliminal presentations in clinically diagnosed Social Anxiety Disorder patients (Amir et al., 2009; Schmidt et al., 2009; Heeren et al., 2011, 2012), as well as in analog samples with moderate to high self-reported social anxiety (Amir et al., 2008; Klumpp and Amir, 2010). The subliminal ABM protocol used in the current study followed the parameters from these previous supraliminal ABM studies, which have demonstrated positive effects of ABM in socially anxious populations (Amir et al., 2008, 2009; Schmidt et al., 2009), but with subliminal presentations. We expected the subliminal ABM protocol to change threat-related attention patterns in accord with the training condition. That is, that participants trained to attend away from threats would show a reduction in threat-related attention bias following ABM. No change in attention pattern was expected in the placebo control condition which was not intended to manipulate attention. We also expected that participants in the ABM condition will display less anxiety and lower stress vulnerability following training relative to participants in the placebo control condition.

## Methods

### Participants

**Figure 1:**
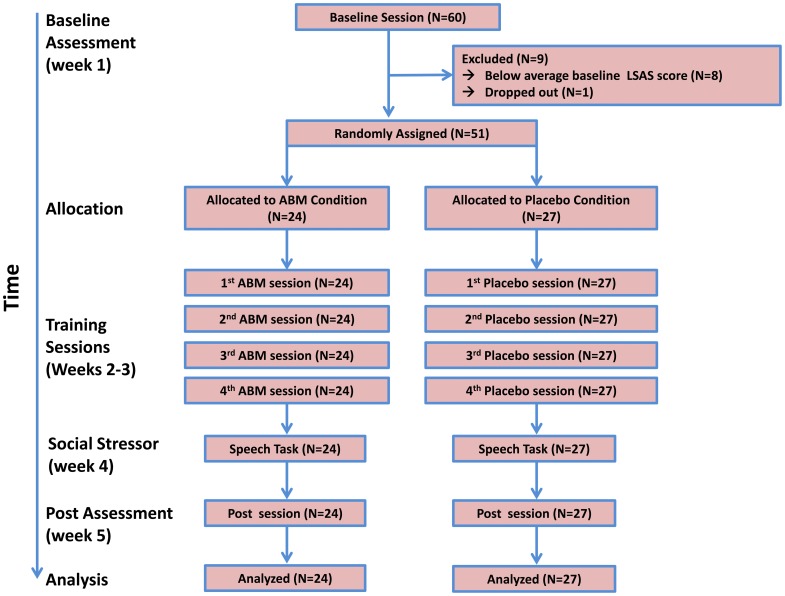
**CONSORT diagram and sequence of events in the study**.

Sixty socially anxious undergraduate students were invited to participate in the study based on their high total scores (>30) on the Liebowitz Social Anxiety Scale (LSAS, Liebowitz, 1987) completed in a mass survey at the beginning of the academic year. A cutoff score of 30 was found to provide the best balance between false positive and false negative diagnostic errors in classifying individuals with social anxiety disorder (Mennin et al., 2002; Rytwinski et al., 2009). The LSAS was again administered to these 60 students in the lab during the pre-assessment session of the study to verify high social anxiety levels. Eight students reported lower levels of social anxiety relative to their initial report and no longer met the criterion of LSAS >30. These students were thus excluded from further participation in the study. An additional student decided not to participate. Thus, 51 participants (mean age = 22.70 years, SD = 1.65; 41 females) were randomly assigned to either an ABM group (*n* = 24) or a placebo control group (*n* = 27). The mean LSAS score for the final sample was 55.73 (*SD* = 16.90), placing their mean score more than 3 standard deviations above the mean for individuals with no axis I diagnosis (Fresco et al., 2001). The groups did not differ in age, gender distribution, baseline threat bias scores, and mean LSAS scores, all *p*s > 0.15 (see Table 1 for baseline means and SDs by group, and Figure 1 for a CONSORT diagram). The study was approved by the institutional review board. Participants provided signed informed consent.

**Table 1:** Means and SDs of baseline, post-training, pre-stressor, and during-stressor measurements by group^*^.

	**Baseline**		**Post-training**	
	**ABM**	**Placebo-Control**	**ABM**	**Placebo-Control**
Gender (F/M)	20/4	20/6		
Age	22.96 (1.94)	22.42 (1.33)		
LSAS score	54.67 (14.55)	55.96 (18.99)	53.63 (16.88)	54.62 (21.28)
STAI-S score	38.00 (10.80)	38.35 (7.92)	34.59 (9.28)	38.54 (9.50)
**DOT-PROBE**				
Mean RT—threat	527 (63)	533 (47)	478 (44)	470 (39)
Mean RT—neutral	527 (58)	532 (50)	479 (40)	469 (33)
Threat bias score	0.10 (19)	−0. 88 (18)	1.01 (18)	−0.97 (17)
**AFFECTIVE SPATIAL CUING**				
Mean RT—threat valid	585 (92)	565 (69)	556 (79)	542 (56)
Mean RT—neutral valid	582 (94)	562 (70)	559 (76)	542 (59)
Mean RT—threat invalid	659 (118)	666 (98)	636 (106)	646 (84)
Mean RT—neutral invalid	675 (146)	664 (92)	635 (102)	647 (89)
Threat engagement	−3.06 (23)	−2.35 (22)	3.59 (22)	−0.49 (20)
Threat disengagement	−16.04 (54)	1.52 (40)	0.74 (36)	−0.83 (37)
	**Pre-stressor**		**During-stressor**	
	**ABM**	**Placebo-Control**	**ABM**	**Placebo-Control**
STAI-S score	38.29 (10.19)	40.27 (10.85)	49.84 (9.75)	52.73 (10.49)

* No between-group differences were found at baseline, post-training, pre-stressor or during-stressor, all ps > 0.10.

LSAS is Liebowitz Social Anxiety Scale, STAI-S is Spielberger State-Trait Anxiety Inventory-State.

### Questionnaires

Social anxiety was assessed with the LSAS (Liebowitz, 1987). This scale consists of 24 items describing social interactions and performance situations. The LSAS possesses strong psychometric properties (Fresco et al., 2001). The Hebrew version of the LSAS was found valid and reliable (Levin et al., 2002). Cronbach's alpha in the current sample was 0.90 and 0.93 for the baseline and post-ABM/Placebo sessions, respectively.

State anxiety was measured with the state sub-scale (STAI-S) of the State-Trait Anxiety Inventory (STAI, Spielberger et al., 1983). The STAI-S consists of 20 items measuring current, situational levels of anxiety. The Hebrew version of the STAI was found valid and reliable (Teichman and Melinic, 1979). Cronbach's alpha in the current sample was 0.89 or higher in each of the STAI-S administrations (baseline, pre and during the stressor task, and at post measurement).

### Attentional bias assessment

#### The dot probe task

**Figure 2:**
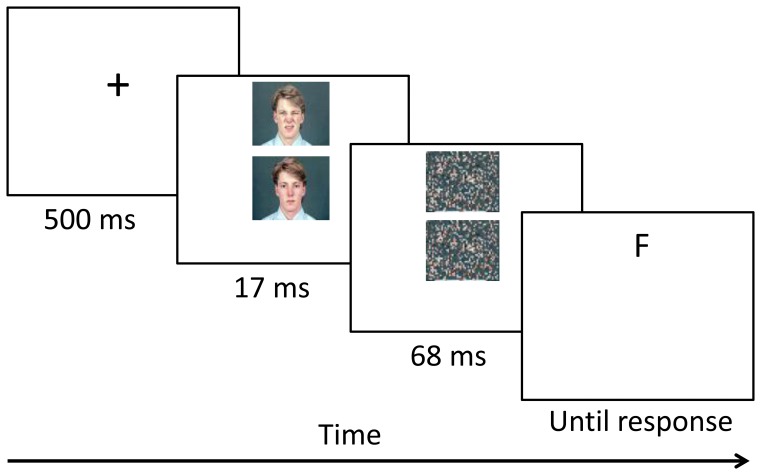
**Sequence of events in a subliminal dot-probe trial**.

The sequence of events on a dot-probe trial is described in Figure 2. Each trial began with the presentation of a fixation display (500 ms; white cross 1 × 1 cm), on which the participants were requested to focus their gaze. The fixation display was followed by a presentation of a pair of faces. Face pairs comprised either disgust-neutral or neutral-neutral facial expressions of the same actor. Pictures of eight different actors were used (four female), taken from a standardized set of emotional expressions (Matsumoto and Ekman, 1988). Each face photograph was placed on a gray square background subtending 50 mm in width and 37.5 mm in height. The face photographs were presented with equal distance from the top and bottom of the fixation cross, with a distance of 15 mm between them. The top photograph was positioned 30 mm from the top edge of the screen. The faces were displayed for 17 ms and were then masked by a pair of scrambled neutral faces displayed for 68 ms (see Mogg and Bradley, 2002 for a similar masking procedure). After the masking disappeared, a target probe consisting of either the letter E or F (font Arial, size 14, bold) appeared at the location previously occupied by one of the masks, and remained on the screen until response. Participants had to determine which of the letters appeared by pressing one of two pre-specified buttons on a mouse. The task comprised 128 trials of disgust-neutral pairs and 32 trials of neutral-neutral pairs, for a total of 160 trials, displayed in a random order.

The 128 disgust-neutral trials were counterbalanced with regard to actor identity, disgusted face location (top, bottom), probe location (top, bottom), and probe type (E, F). Attention bias for each participant was calculated by subtracting the mean RT of trials in which the target probe appeared at the disgust face location from the mean RT of trials in which the target appeared at the neutral face location. Positive scores reflect a bias toward threat (threat vigilance), whereas negative scores reflect an attentional bias away from threat (threat avoidance). On neutral–neutral trials target location and type were fully counterbalanced and RTs from these trials were not included in attention bias calculation.

#### The affective spatial cueing task

Assessing attention bias using the dot-probe task following ABM may reflect only near transfer of the training effect since it relies on the same task demands and stimuli as the ABM training itself. To further test for generalization of potential changes in threat attendance as a function of ABM, we used an affective variant of Posner's spatial cueing task (Stormark et al., 1995; Fox et al., 2001, 2002). In Posner's original task (Posner, 1980), a cue appears in one of two locations, and is followed by a target at the cued location on a majority of the trials (valid cue) and at the alternative location on a minority of the trials (invalid cue). Speeding on valid trials is attributed to the benefits of attentional engagement with the cued location. Slowing on invalid trials is associated with the costs of having to disengage attention from the cued location. Systematic manipulation of the emotional content of cues reveals the effect of cue valence on attention. Studies using this task typically report increased dwelling time on threat invalid cues relative to neutral invalid cues, in anxious relative to non-anxious individuals (Bar-Haim et al., 2007). This is thought to reflect a difficulty in disengaging attention from threat among anxious individuals. To test for far transfer of training effects we used the emotional spatial cuing task in addition to the dot-probe task and also used different stimuli (angry faces rather than disgusted faces). Both disgust and anger expressions constitute threatening cues to socially anxious individuals, and attentional vigilance for both types of stimuli was demonstrated in this population (e.g., Mogg et al., 2004; Pishyar et al., 2004). Here, the emotional cues consisted of face photographs of 16 different actors (8 females) taken from the NimStim stimulus set (Tottenham et al., 2009). Two pictures of each actor were selected depicting an angry and a neutral expression. The target was an arrow pointing either up or down. Participants had to determine the arrow's direction by pressing one of two pre-specified buttons on a mouse. Cue and target stimuli were presented inside two dark gray boxes (50 mm × 65 mm) which were displayed continuously to the left and the right of the screen center. Each trial was initiated by a fixation cross presented in the center of the screen for 500 ms. Then, the cue was presented either in the left or right box for 17 ms, and immediately masked by a scrambled neutral face displayed for 68 ms. The target arrow then appeared in either the same box as the cue (valid trials) or the opposite box (invalid trials) and remained on the screen until response. The task comprised 192 trials of which 75% were valid and 25% were invalid. Within each type of trial (valid/invalid), cue type (neutral/angry), target location (left/right), and target type (pointing up/pointing down) were fully counterbalanced. Throughout the task each actor's photographs appeared a total of 12 times—6 times with an angry expression and 6 times with a neutral expression. Threat engagement was calculated as mean RT for valid neutral trials minus mean RT for valid threat trials. Positive engagement scores reflect attentional bias toward threat (threat engagement), whereas negative engagement scores reflect an attentional bias away from threat (threat avoidance). Threat disengagement was calculated as mean RT for invalid threat trials minus mean RT for invalid neutral trials. Positive disengagement scores are considered to reflect a difficulty in disengaging attention from threat stimuli.

### Attention bias modification (ABM)

The ABM version of the dot-probe task displayed the same stimuli as those used for threat bias assessment except that target probes (E, F) appeared only at the location previously occupied by neutral faces with the aim of implicitly establishing these as a predictive cue for the location of the probe. The placebo control group received the same number and type of trials as the ABM group but in a fully counterbalanced manner as was done during threat bias assessment. Thus, no attention modification was expected in the placebo control group.

### Visual masking efficacy test

To ensure that participants were not consciously aware of the emotional valence of the masked faces, an objective detection task was used (Merikle et al., 2001). This two-alternative forced choice task comprised 32 trials. Stimuli were pairs of identical face pictures (i.e., two neutral faces or two disgusted faces) taken from those presented in the assessment version of the dot-probe task. In each trial, a face pair was presented and masked in the same manner as in the dot-probe task. Participants were told that half of the trials contain a pair of identical faces featuring a negative valence, whereas the other half contains a pair of identical neutral faces, and had to indicate via button press whether the faces in each trial were “neutral” or “negative.” A 95% confidence interval was calculated to reflect chance level performance.

### Social stress induction task

The social stress induction task was similar to the one used by Amir et al. (2008). Participants were asked to choose one of three discussion topics (using nuclear energy to produce electricity, school uniform, or toll roads) and prepare a 5-minute speech concerning claims in favor of and against the selected topic. Participants were informed that their speech would be videotaped and later evaluated for quality by the research staff. During the speech task an unfamiliar male experimenter was present in the room, provided instructions, and operated the video camera.

### Procedure

Over a period of five weeks, participants completed a baseline assessment session, four attention training/placebo sessions, a stress induction session, and a final evaluation session (see CONSORT diagram Figure 1). The baseline assessment session lasted 25 min during which participants completed the STAI-S, LSAS, and the dot-probe and affective spatial cueing tasks. All computerized tasks were run in a darkened room, on a 17-inch-screen laptop computer (Lenovo R61i), using E-Prime software. Participants were seated at a viewing distance of 80 cm from the monitor. Following the assessment session participants received four training sessions according to their group assignment (two sessions on nonconsecutive days per week, over two weeks). Each training session lasted approximately 10 min. The sixth session, conducted 4–7 days following the last training session, was dedicated to testing the effects of ABM on stress vulnerability using the social stress induction task. This session took place in a different room than the room of the training sessions. The male experimenter administering this session was unfamiliar to the participants and blind to all aspects and purposes of the study. Participants completed the STAI-S in a waiting room. The experimenter then invited participants to enter the testing room where the social stress procedure was conducted. Following 5 min of speech preparation participants were asked to step up to a marked spot in front of the camera and deliver their speech. Two minutes into their speech, the experimenter temporarily paused the task and asked participants to complete the STAI-S again. The experimenter made it clear that the speech will be resumed shortly after completion of the questionnaire. Following one additional minute of speech participants were halted and thanked. Stress vulnerability was indexed as the change between pre- and during-stressor STAI-S. The seventh and final session was held in the following week and took place in the same room as the training sessions. Each participant performed the same dot-probe and affective spatial cueing tasks as in the baseline session. Then, participants completed the test of visual masking efficacy followed by completion of the LSAS and the STAI-S.

### Data analysis

Trials with RTs shorter than 150 ms or longer than 2000 ms, or incorrect response were excluded. Then, for each participant, mean RT per trial type was calculated, and trials with RTs deviating by more than 2.5 SDs from the mean were further excluded. This resulted in the removal of an average of 6% of all trials per participant.

The effect of subliminal ABM on attention was assessed for bias scores on the dot-probe task and for RTs in the affective spatial cueing task. Dot-probe attention bias scores were subjected to a 2 × 2 repeated-measures ANOVA with Group (ABM, placebo) as a between-subjects factor and Time (baseline, post-training) as a repeated within-subject factor. Response times on the affective spatial cueing task were submitted to a 2 × 2 × 2 × 2 ANOVA with Group (ABM, placebo) as a between-subjects factor, and Time (baseline, post-training), Cue Validity (valid, invalid), and Cue Valence (threat, neutral) as repeated within-subject factors.

To examine the effect of subliminal ABM on trait social anxiety levels, total social anxiety scores from the LSAS were submitted to a 2 × 2 ANOVA with Group (ABM, placebo) as a between-subjects factor and Time (baseline, post-training) as a repeated within-subject factor. To examine the effect of subliminal ABM on vulnerability to social stress, a repeated-measures ANOVA was conducted on STAI-S scores before and during- the stressor task. Group (ABM, placebo) served as a between-subjects factor and Stressor-Phase (pre-stressor, during-stressor) served as a repeated within-subject factor.

Because recent studies suggest that baseline attention bias toward threat may predict supraliminal ABM training efficacy (Amir et al., 2011), as well as cognitive-behavioral treatment efficacy (Waters et al., 2012), we explored this possibility in the current subliminal ABM study. We conducted two *post-hoc* analyses to test whether baseline vigilance or avoidance (attention bias toward or away from threat) modulated the effect of subliminal ABM on social anxiety and stress vulnerability. First, following Waters et al. (2012), we divided the participants to two groups based on whether they had a bias toward threat (“attenders,” attention bias > 0; *n* = 29) or a bias away from threat (“avoiders,” attention bias < 0; *n* = 21) at baseline. This new dichotomous variable was entered as an additional between-subjects factor in the above described primary ANOVAs. Second, following Amir et al. (2011), we regressed baseline attention bias as a continuous predictor, along with training group (ABM/Placebo) (step 1) and their interaction term (step 2) on state anxiety (STAI-S) change score from pre- to during the stress induction episode. The same regression model was also applied to change in trait social anxiety (LSAS) from pre- to post ABM/Placebo.

## Results

### Visual masking efficacy test

All participants but one performed the task at chance level (mean = 50.12% correct, *SD* = 8.12), indicating that the masking procedures were effective and that participants were unaware of the affective valence of the faces. One participant had an above-threshold accuracy performance (72% correct). All the analyses reported exclude the data from this participant. When analyses were conducted including this particular subject, no changes were noted in the results pattern.

### Baseline measurements

Means and SDs for LSAS, STAI-S, and RTs and bias scores on the dot-probe and affective spatial cueing tasks at baseline by training condition are provided in Table 1 (left panel). None of these measures significantly differed between the ABM and Placebo-Control groups (all *p*s > 0.19). Attention bias in the dot probe task as well as engagement and disengagement biases in the affective spatial cueing task were not significantly different than zero neither in the ABM group nor in the Control group (all *p*s > 0.15).

### Post-training measurements

Means and SDs for LSAS, STAI-S, and RTs and bias scores on the dot-probe and affective spatial cueing tasks at post-training by training condition are provided in Table 1 (right panel).

#### Change in attention threat bias

***Dot-probe.*** This analysis yielded no significant main or interaction effects, indicating no detectable changes in attention bias scores from pre- to post-training, all *p*s > 0.68.

***Affective spatial cuing task.*** RTs to invalidly cued trials were longer than RTs to validly cued trials reflecting the classic Posner validity effect, *F*_(1, 48)_ = 187.13, *p* < 0.0001, η^2^_*p*_ = 0.80. In addition, a main effect of Time was found, reflecting faster overall RTs following ABM/Placebo, *F*_(1, 48)_ = 6.39, *p* < 0.05, η^2^_*p*_ = 0.12. No other main or interaction effects reached statistical significance.

#### Trait social anxiety (LSAS)

This analysis yielded no significant main or interaction effects, indicating that subliminal ABM did not affect self-reported trait social anxiety (LSAS), all *p*s > 0.41.

#### Social stress vulnerability

A main effect of Stressor-Phase was found, *F*_(1, 48)_ = 54.86, *p* < 0.0001, η^2^_*p*_ = 0.53, demonstrating that the stressor task significantly increased state anxiety levels from pre-stressor (mean = 39.32, *SD* = 10.48) to during-stressor (mean = 51.34, *SD* = 10.14). No other main or interaction effects reached statistical significance, all *p*s > 0.32.

### Secondary *post-hoc* analyses—testing the effect of baseline attention bias

#### Baseline bias as a dichotomous factor

Means and SDs of all baseline and post-training measurements for baseline attenders and avoiders are presented in Table A1 (see appendix). The average baseline attention bias scores in the attenders (12 ms, *SD* = 9) and the avoiders (−18 ms, *SD* = 12) groups were each significantly different from zero, *t*_(28)_ = 7.44, *p* < 0.0001 and *t*_(20)_ = −6.63, *p* < 0.0001, respectively. There were no significant differences in baseline bias scores between ABM and control participants neither in the attenders nor in the avoiders groups (all *p*s > 0.6, for means and SDs see Table 2).

**Table 2:** **Means and SDs of baseline attention bias and state anxiety (STAI-S) pre- and during-stressor, for baseline attenders and avoiders by training group**.

	**Baseline attenders**		**Baseline avoiders**	
	**ABM (*N* = 15)**	**Placebo-Control (*N* = 14)**	**ABM (*N* = 9)**	**Placebo-Control (*N* = 12)**
Baseline attention bias	12 (8)	13 (10)	−19 (16)	−17 (9)
STAI-S				
Pre-Stressor	39.33 (7.91)	36.65 (8.26)	36.56 (13.55)	44.50 (12.27)
During-Stressor	47.60 (9.72)	54.71 (9.93)	53.57 (9.12)	50.42 (11.07)

Adding the baseline attention bias (toward or away from threat) as a factor to all analyses produced two significant interaction effects: first, when testing for effects of ABM on attention bias using the dot-probe task, a Time-by-Baseline Bias interaction effect emerged, *F*_(1, 46)_ = 24.27, *p* < 0.0001, η^2^_*p*_ = 0.35. Both participants who had an attention bias toward threat and participants who had an attention bias away from threat at baseline (means = 12.31 and −17.97, *SD*s = 8.91 and 12.42, respectively) converged toward having no bias following ABM/Placebo (means = 0.44 and −0.66, *SD*s = 18.43 and 15.13, respectively).

**Figure 3:**
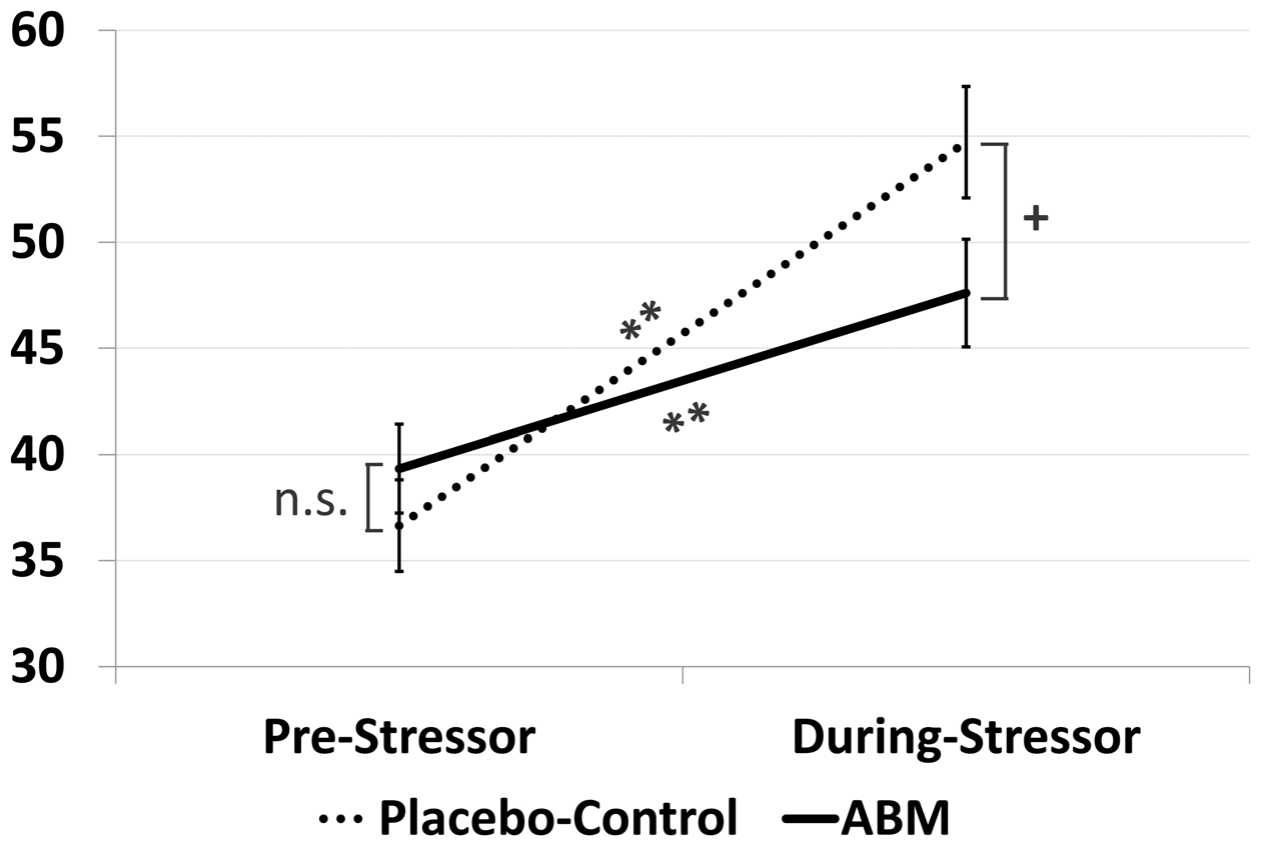
**State Anxiety Scores (STAI-S) and standard error bars for participants with baseline threat vigilance pre- and during the social stressor task by attention training condition.** ^**^p < 0.0001; + *p* = 0.06.

The second significant interaction was related to the effect of ABM on stress vulnerability. STAI-S means and SDs before and during the social stressor task are presented in Table 2. There was a significant three-way Stressor-Phase-by-Group-by-Baseline Bias interaction effect, *F*_(1, 46)_ = 12.31, *p* < 0.001, η^2^_*p*_ = 0.21. To explicate this interaction, two ANOVAs of Group (ABM, placebo) by Stressor-Phase (pre-stressor, during-stressor) were conducted, one for participants who had attention bias away from threat at baseline (avoiders) and one for participants who had attention bias toward threat at baseline (attenders). For threat avoiders, those who received ABM showed larger elevation in state anxiety in response to the stressor task relative to their counterparts in the placebo training group. However, this interaction was non-significant, *F*_(1, 19)_ = 3.89, *p* = 0.063. For threat attenders, however, a significant Stressor-Phase-by-Group interaction effect was found, *F*_(1, 27)_ = 10.36, *p* < 0.005, η^2^_*p*_ = 0.28. Follow-up contrasts indicated that both groups (ABM and Placebo) showed significant increases in state anxiety from before-to-during stressor, both *p*s < 0.001. Additional between-group contrasts revealed that the two groups did not differ on STAI-S scores before stress induction, *t*_(27)_ = −0.89, *p* = 0.38. Interestingly, the ABM group showed lower STAI-S scores relative to the placebo group during stress, *t*_(27)_ = 1.95, *p* = 0.062. This non-significant trend may suggest that among those who attended toward threat at baseline, those who received ABM were less vulnerable to the stressor (Figure 3).

#### Baseline bias as a continuous factor

**Figure 4:**
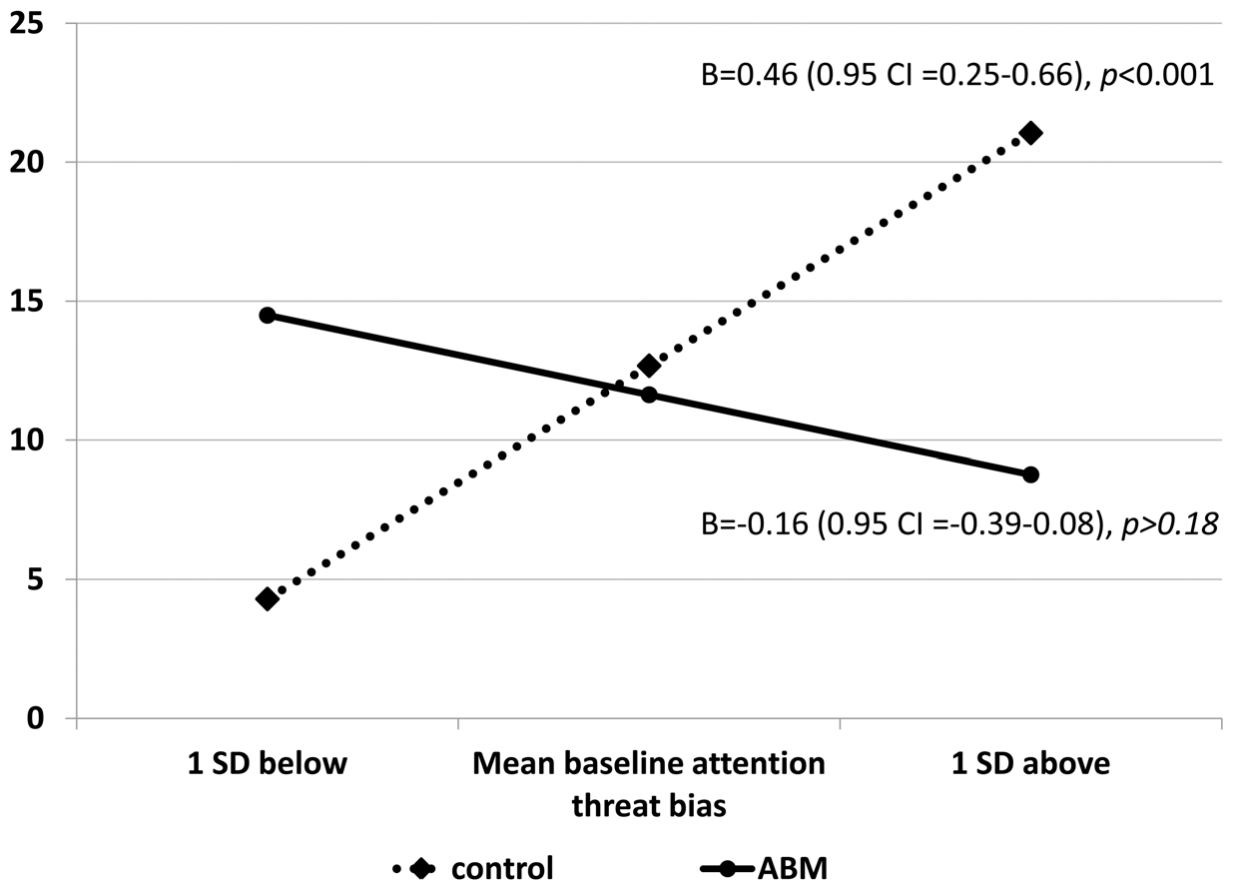
**Simple slope analyses: the estimated change in state anxiety from pre to during the stressor task, as a function of the interaction between baseline attention bias and training group (ABM/Placebo-control)**.

The estimated coefficients and significance levels for the two steps in the regression model are shown in Table 3. The overall regression model significantly explained 31 percent of the variance in state anxiety change due to stress induction, *F*_(3, 46)_ = 6.80, *p* < 0.001. This model explained significantly and substantially more variance in stress-related anxiety change as compared to the model considering only baseline attention bias and group as single predictors, without taking into account their interaction. Specifically, when not considering the interaction in the model, ABM/Placebo Group did not predict state anxiety change. Baseline attention bias predicted state anxiety change at a trend level of significance with greater baseline threat bias predicting greater elevations in state anxiety following stress induction. Importantly, the interaction term between baseline attention bias and ABM/Placebo group significantly predicted state anxiety change. Follow-up simple slope analyses demonstrated that for the Placebo-Control group the slope coefficient was positive and significantly different from zero, *B* = 0.46, *t*_(24)_ = 4.55, *p* < 0.0001, suggesting that in this group, individuals with greater attention bias to threat at baseline demonstrated larger elevations of anxiety during the stress task. In contrast, for the ABM group the slope coefficient was not significantly different from zero, *B* = −0.16, *t*_(22)_ = −1.36, *p* > 0.15 (Figure 4).

**Table 3:** **Estimated coefficients, standard errors, and 0.95 confidence intervals for predictors in the two steps of the regression model predicting stressor-related anxiety change**.

	**Predictor**	**B**	**SE**	***t***	**95% CI**	***R*^2^**	**Δ*R*^2^**
Step 1	Baseline attention bias	0.15^+^	0.09	1.74	−0.02 – 0.33	0.06	
	Training group	−1.06	3.18	−0.34	−7.45 – 5.33		
Step 2	Baseline attention bias	0.46^**^	0.11	4.27	0.24 – 0.67	0.31	0.25^**^
	Training group	−1.30	2.76	−0.47	−6.85 – 4.26		
	Baseline attention bias × Training group	−0.61^**^	0.15	−4.04	−0.92 – −0.31		

+ p = 0.089;

** p < 0.001. Training group = ABM/Placebo-control groups. B = unstandardized estimated coefficient. SE = standard error. CI = confidence interval.

The regression model predicting change in trait social anxiety (LSAS) from pre- to post-training was non-significant (*p* > 0.8).

## Discussion

The present study is the first to report a randomized controlled ABM trial using subliminally-presented stimuli in high socially-anxious individuals. The aim of the study was to examine whether subliminal ABM training away from threat faces was effective in reducing levels of social anxiety and social stress vulnerability in socially-anxious students. In our effort to accomplish this aim, we relied on commonly used ABM and attention bias measurement methods that have proved effective in supraliminal ABM research in similar populations. However, despite this methodological effort and in contrast to our expectations, the subliminal ABM used in the current study did not induce detectable change in threat-related attention, neither on the dot-probe task (near transfer) nor on the affective spatial cueing task (far transfer). We were also unable to find an effect of ABM on self-reported trait social anxiety.

There are various potential explanations for the null-findings which could be broadly classified into four general types: (a) that subliminal ABM is inherently ineffective for changing preconscious attention patterns in anxious individuals; (b) that the specific subliminal ABM protocol used here was not effective in inducing the expected change in attention patterns; (c) that subliminal ABM did in fact modify attentional patterns, but our measurement precision failed to detect this change; and (d) that subliminal attention processes are related to anxiety only in a sub-group of socially anxious individuals, those who demonstrate threat-related attention bias at baseline. Because our sample as a whole did not demonstrate a measureable attention bias toward subliminal threat at baseline this possibility was tested in *post-hoc* analyses looking at the role of baseline threat bias in anxiety reduction as a function of ABM. The theoretical and practical implications of each of the above-listed options are distinct in important ways. Next we discuss the implications of each of these possibilities.

Subliminal ABM could be inherently ineffective in inducing change in preconscious attention patterns because the relevant neuro-cognitive mechanisms supporting this process might be less malleable to change by ABM as compared to processes occurring within perceptual awareness. Specifically, subliminal threat detection typically involves sub-cortical structures such as the amygdala, one of the core components in danger detection and evaluation (Ledoux, 2000; Amaral, 2002), whose function relies, at least in part, on automatic, rapid-responding neural architecture (Ohman and Mineka, 2001; Ohman, 2005; Adolphs and Spezio, 2006). If preconscious attention patterns are indeed less malleable to change, two important conclusions may be derived: practically, there may be no reason to invest further efforts in subliminal ABM methods. Theoretically, one may speculate that consistent failure to modify attentional patterns using subliminal ABM is consistent with the notion that modification of threat bias by ABM and the associated reduction in anxiety are mediated by later processes of attention control rather than by automatic attention capture (Browning et al., 2010; Eldar and Bar-Haim, 2010; Heeren et al., 2013).

A second possible explanation for the current null results is that the specific subliminal ABM protocol used here was not sufficiently effective to induce the expected change in attention patterns. For example, it might be that trying to change early preconscious processes using subliminal ABM requires more training sessions, more trials per session, different stimuli, or different masking procedure. If so, more experimental research is needed to unveil these parameters that individually or together obscure the expected effects.

Third, it may be considered that the subliminal ABM training did in fact influence attentional patterns, but our measurement tools failed to detect this change. This could be due to the relatively long time interval that elapsed from the end of training to the post-training measurement of attention bias, or due to the effects of using subliminal stimuli. For example, one may consider the possibility that consciously perceived threat is necessary for ABM effects on attention to surface in measurement. In supraliminal ABM protocols the presence of threat stimuli is consciously perceived throughout all assessment and practice sessions. In contrast, in the current study conscious perception of threat never occurred neither during training nor during threat bias assessments. Future studies may consider measuring change in threat bias using supraliminal presentations even when ABM is subliminally delivered.

Finally, the finding of reduced stress vulnerability following subliminal ABM in participants who demonstrated attention bias toward threat at baseline may offer a clue that for a sub-group of participants there was in fact some effect of subliminal ABM on stress vulnerability. This finding is in accord with recently reported results from supraliminal ABM in patients diagnosed with general social phobia (Amir et al., 2011). In Amir et al. (2011), patients in the ABM condition who had greater threat bias at baseline displayed significantly larger reductions in clinician-rated social anxiety symptoms relative to their counterparts in the placebo condition. ABM did not differ from placebo in patients who did not show threat bias at baseline. These findings from Amir et al. (2011) and the present finding are also in line with the basic rationale for ABM procedures. That is, that pre-treatment threat bias is the target for ABM; hence the absence of such bias might render ABM ineffective. A similar rationale was offered in a randomized controlled ABM study with clinically anxious children, which applied a baseline bias toward threat as an inclusion criterion for participation (Eldar et al., 2012). The current finding along with previous results highlights the possibility that ABM procedures may be beneficial to a specific sub-group of socially-anxious individuals characterized by attention bias toward threatening cues at baseline. If proved reliable, such specificity in predicting treatment efficacy may be ultimately applied to personalize anxiety treatment. However, it is important to keep in mind that, at least in the present study, this finding was part of a *post-hoc* exploration and could be merely incidental. It is also important to note that attention bias in both threat attenders and threat avoiders converged toward zero at post-training, thus could simply reflect regression to the mean. Future studies may benefit from designs that specifically and a priori hypothesize about the role of baseline threat bias in the clinical response to ABM.

Despite the discouraging findings with subliminal ABM thus far, the potential of this intervention should not be dismissed prematurely. If future studies could substantiate evidence that subliminal training of threat-related attention may have anxiolytic effects, it would point to the potential of ABM to target components of threat processing that function outside perceptual awareness. Such specific processes may occur independently and within different brain networks than processes activated by consciously-perceived threats (Etkin et al., 2004; Li et al., 2007). Future studies may utilize brain imaging techniques to directly examine and compare the underlying neuro-cognitive mechanisms affected by subliminal and supraliminal ABM. If indeed subliminal and supraliminal ABM methods influence different neuro-cognitive mechanisms related to anxiety, they may possess additive therapeutic values, and may prove more efficient if delivered as a combined treatment procedure. This possibility should be determined in future research directly comparing subliminal ABM, supraliminal ABM, and a combination of the two.

Interpretation of the results of the present study should be considered in light of important limitations. First, the participants in the present study were not clinically-diagnosed with social phobia, but rather represent a sample of undergraduate students who self-reported high levels of social anxiety. While the use of analog populations typically provides an opportunity to test preliminary treatment-related ideas, in the current study it might have also limited the potential to detect anxiety-related effects of subliminal ABM training. Future studies with clinically-diagnosed populations could further test the efficacy of subliminal ABM. Second, the present sample may not be large enough to detect existing effects if these are relatively small. It should be noted, however, that the detected effect of subliminal ABM on social stress vulnerability within the threat-attenders sub-group is quite robust considering the small sample size. Third, the fact that attention bias toward subliminal threat was not significantly different from zero in the current sample could be considered a limitation that may have hampered the possibility to detect attentional and anxiety-related changes. Considering the small-to-medium effect size of the threat bias phenomenon in general, it is not uncommon that a single study will not be able to find anxiety-related attention bias (Bar-Haim et al., 2007). Furthermore, the effect is many times reported for between group designs comparing anxious individuals to non-anxious controls, while in the current study there were only high-anxious participants. One way to probe this shortcoming is to analyze the results referring to participants who actually showed threat bias at baseline (as was done here using *post-hoc* analyses). While these analyses seem to support the notion that baseline threat bias may be important for ABM success, caution should be taken in the interpretation of these *post-hoc* findings, particularly those based on the dichotomous split to attenders and avoiders. This particular analysis relied on very small group sizes and also suffers from the possibility that some group members may not truly deviate from zero bias. These concerns may be alleviated to some extent by the supportive findings relying on baseline attention bias as a continuous variable in the regression analyses and should be explored in future research.

In conclusion, the current study is mainly offering null results of subliminal ABM, as we were unable to show direct effects of subliminal ABM training on attention patterns or anxiety levels. Nevertheless, we think it may be important for the ABM research community to be exposed to these findings so that both an open discussion of the issue could be advanced and future studies could use this failure as a stepping stone for their ABM designs. The current study is the first to report null results for subliminal ABM conducted with an anxious population and it corresponds with recently published null effects of supraliminal ABM (Carlbring et al., 2012; Bunnell et al., 2013; Neubauer et al., 2013). We hope that researchers would continue to share both null- and positive-findings concerning ABM in order to advance understanding and experimentation in this field. In the same vein, we also thought it is worthwhile to report the *post-hoc* analyses suggesting that subliminal ABM training may carry some potential to reduce social stress vulnerability, and that baseline threat bias may serve as a marker for such efficacy.

## Conflict of interest statement

The authors declare that the research was conducted in the absence of any commercial or financial relationships that could be construed as a potential conflict of interest.

## Appendix

**Table A1:** **Means and SDs of baseline, post-training, pre-stressor, and during-stressor measurements for baseline attenders and avoiders by training group**.

	**Baseline attenders**			**Baseline avoiders**		
	**ABM**	**Placebo-Control**	**Total**	**ABM**	**Placebo-Control**	**Total**
	**(*N* = 15)**	**(*N* = 14)**	**(*N* = 29)**	**(*N* = 9)**	**(*N* = 12)**	**(*N* = 21)**
Gender (F/M)	12/3	12/2	24/5	8/1	8/4	16/5
Age	22.93 (1.83)	22.21 (1.31)	22.59 (1.62)	23 (2.24)	22.67 (1.37)	22.81 (1.75)
**BASELINE MEASUREMENTS**						
LSAS score	54.60 (12.59)	58.71 (22.10)	56.59 (17.62)	54.78 (18.19)	52.75 (14.89)	53.62 (15.98)
STAI-S score	36.07 (7.25)	39.57 (7.94)	37.76 (7.66)	41.22 (15.00)	36.92 (7.99)	38.76 (11.39)
Dot-probe						
Mean RT—threat	517 (65)	531 (40)	524 (54)	543 (61)	535 (56)	538 (57)
Mean RT—neutral	529 (66)	544 (45)	536 (56)	524 (48)	517 (54)	520 (50)
Threat bias score	11.54 (8)	13.13 (10)	12.31 (9)	−18.96 (16)	−17.22 (9)	−17.97 (12)
Affective spatial cuing						
Mean RT—threat valid	575 (100)	569 (69)	572 (85)	603 (80)	559 (70)	578 (76)
Mean RT—neutral valid	580 (105)	565 (69)	573 (88)	586 (79)	560 (74)	571 (75)
Mean RT—threat invalid	651 (129)	663 (90)	656 (110)	673 (103)	669 (111)	671 (105)
Mean RT—neutral invalid	671 (156)	656 (83)	664 (125)	682 (137)	674 (104)	677 (116)
Threat engagement	5.31 (23)	−4.47 (20)	0.59 (22)	−17.00 (14)	0.12 (24)	−7.22 (22)
Threat disengagement	−20.46 (43)	6.98 (44)	−7.22 (44)	−8.67 (70)	−4.85 (37)	−6.49 (52)
**STRESS-RELATED MEASUREMENTS: STAI-S**						
Pre-stressor	39.33 (7.91)	36.65 (8.26)	38.04 (8.05)	36.56 (13.55)	44.50 (12.27)	41.09 (13.13)
During-stressor	47.60 (9.72)	54.71 (9.93)	51.03 (10.30)	53.57 (9.12)	50.42 (11.07)	51.77 (10.16)
**POST-TRAINING MEASUREMENTS**						
LSAS Score	55.13 (17.89)	55.64 (23.19)	55.38 (20.24)	51.11 (15.77)	53.42 (19.76)	52.42 (17.77)
STAI-S score	33.60 (7.07)	36.07 (7.63)	34.79 (7.32)	36.23 (12.45)	41.42 (10.92)	39.20 (11.60)
Dot-probe						
Mean RT—threat	470 (46)	470 (27)	470 (37)	491 (38)	470 (51)	479 (46)
Mean RT—neutral	471 (39)	470 (25)	470 (32)	491 (40)	468 (42)	478 (42)
Threat bias score	1.14 (20)	−0.31 (18)	0.44 (18)	0.79 (14)	−1.75 (16)	−0.66 (15)
Affective spatial cuing						
Mean RT—threat valid	544 (58)	543 (49)	544 (53)	575 (107)	541 (67)	556 (86)
Mean RT—neutral valid	546 (65)	545 (50)	545 (57)	582 (92)	539 (70)	557 (81)
Mean RT—threat invalid	622 (92)	634 (80)	628 (85)	658 (129)	660 (91)	659 (106)
Mean RT—neutral invalid	618 (90)	633 (69)	625 (79)	663 (119)	664 (109)	664 (111)
Threat engagement	1.47 (22)	1.09 (23)	1.29 (22)	7.12 (24)	−2.33 (16)	1.72 (20)
Threat disengagement	4.41 (39)	1.48 (35)	2.99 (36)	−5.37 (32)	−3.51 (41)	−4.31 (36)
